# Understanding fatigue in progressive supranuclear palsy

**DOI:** 10.1038/s41598-021-96443-z

**Published:** 2021-08-19

**Authors:** Jong Hyeon Ahn, Joomee Song, Dong Yeong Lee, Jinyoung Youn, Jin Whan Cho

**Affiliations:** 1 Department of Neurology, Samsung Medical Center, Sungkyunkwan University School of Medicine, 81 Irwon-ro, Gangnam-gu, Seoul, 06351 Republic of Korea; 2 Neuroscience Center, Samsung Medical Center, 81 Irwon-ro, Gangnam-gu, Seoul, 06351 Republic of Korea

**Keywords:** Movement disorders, Neurodegenerative diseases

## Abstract

Fatigue is a common and disabling non-motor symptom (NMS) of Parkinson’s disease (PD); however, it has been poorly understood in patients with progressive supranuclear palsy (PSP). We investigated the association between fatigue, clinical features, and other NMS in patients with probable PSP. In 72 probable PSP patients, fatigue was investigated using the Parkinson Fatigue Scale (PFS). Further, all patients were evaluated using the PSP rating scale (PSPRS), Beck Depression Inventory (BDI), Mini-Mental State Examination (MMSE), Frontal Assessment Battery (FAB), PD Sleep Scale (PDSS), NMS scale (NMSS), PD Questionnaire-39 summary index (PDQ-39 SI), and Scale for outcomes in PD-Autonomic (SCOPA-AUT). The prevalence of fatigue assessed by PFS was 38.9% (28/72) in patients with PSP. The secondary fatigue was defined as fatigued patients with depression and/or sleep disturbances. We divided the patients into primary (n = 15), secondary (n = 13), and non-fatigue groups. There were no differences in age, sex, disease duration, and PSPRS, PDSS, MMSE, and FAB scores among the three groups. The primary fatigue group had higher scores in PDQ-39 SI compared to the non-fatigue group. The secondary fatigue group showed higher scores in NMSS, PDQ-39 SI, and SCOPA-AUT compared to the non-fatigue group. PFS was positively correlated with NMSS and PDQ-39 SI and SCOPA-AUT. Fatigue is common in patients with PSP and is associated with the NMS and the quality of life in these patients. The present study provides meaningful insight into fatigue in patients with PSP.

## Introduction

Progressive supranuclear palsy (PSP) is a rapidly progressive neurodegenerative disease characterized by supranuclear gaze palsy, frequent falling, and parkinsonism^1^. Patients with PSP not only have motor symptoms but also exhibit various non-motor symptoms (NMS), such as cognitive decline, urinary symptoms, emotional problems, and fatigue^2^. Fatigue is a disabling symptom in various movement disorders including Parkinson’s disease (PD), and studies showed that fatigue significantly affects the patients’ quality of life (QoL)^3–5^. Several studies have investigated NMS including fatigue in patients with PSP and reported that sleep/fatigue problems are common in these patients, with a prevalence of 82.9–100%^2,6–9^. However, none of the previous studies used the specialized scale to assess fatigue, and their results were based on the non-motor symptoms scale for PD (NMSS). The NMSS contains a subdomain for evaluating sleep/fatigue; however, it is difficult to precisely measure fatigue and its severity because only one of the four questions included in this domain is related to fatigue (does fatigue or lack of energy limit the patients’ daytime activities?)^10^. Generally, fatigue is described as an overwhelming feeling of tiredness and exhaustion occurring in the context of various neurological diseases^3^. Fatigue has been inconsistently used in previous studies, and it is difficult to define fatigue because it is subjective and various factors such as mood disorders, sleep disorders, orthostatic hypotension, and anemia can affect fatigue^4,5^.

Thus, fatigue is a common NMS in patients with PSP. Specialized studies focused on fatigue and investigation of its clinical impact are needed to improve the understanding and management of this disabling symptom. Herein, we investigated fatigue in patients with probable PSP using the Parkinson Fatigue Scale (PFS), which is a specialized scale for assessing fatigue^5^. Moreover, we also investigated the associated clinical features, including other NMSs.

## Results

### Demographic characteristics, clinical features, and factors associated with fatigue in patients with PSP

In the present study, 72 patients with probable PSP were included (62 patients with PSP with Richardson’s syndrome [PSP-RS], eight patients with PSP with predominant parkinsonism, one patient with PSP with progressive gait freezing, and one patient with PSP with predominant frontal presentation). The demographic characteristics of the enrolled patients are shown in Table 1. The prevalence of fatigue assessed by PFS was 38.9% (28/72) in patients with PSP. We divided the patients into primary fatigue, secondary fatigue, and non-fatigue groups^11–13^. Fifteen patients (15/28, 53.6%) were included in the primary fatigue group and thirteen patients (13/28, 46.4%) were included in the secondary fatigue group (Table 1).

**Table 1:** Demographics and clinical characteristics.

	Total (n = 72)	Primary fatigue (n = 15)	Secondary fatigue (n = 13)	Non-fatigue (n = 44)	*p* value	*p* value (primary vs. non-fatigue)	*p* value (primary vs. secondary fatigue)	*p* value (secondary vs. non-fatigue)
Age	69.5 ± 7.8	72.9 ± 5.1	69.5 ± 7.5	68.4 ± 8.5	0.154	–	–	–
Sex (male, %)	45 (62.5)	13 (86.7)	7 (53.8)	25 (56.8)	0.093	–	–	–
Disease duration (m)	44.6 ± 33.0	57.4 ± 35.3	39.5 ± 33.4	41.8 ± 31.7	0.238	–	–	–
Diagnosis (PSP-RS/PSP-P/PSP-PGF/PSP-F)	(62/8/1/1)	(12/2/0/1)	(11/2/0/0)	(39/4/1/0)	0.546	–	–	–
PFS	42.4 ± 20.8	3.9 ± 0.5	4.1 ± 0.5	28.2 ± 13.8	< 0.001	< 0.001	> 0.999	< 0.001
**PSPRS**						–	–	–
History	6.2 ± 3.8	6.4 ± 3.5	7.8 ± 5.0	5.6 ± 3.4	0.179	–	–	–
Mentation	1.8 ± 2.3	1.9 ± 2.1	2.8 ± 2.9	1.4 ± 2.1	0.157	–	–	–
Bulbar	2.5 ± 1.7	2.6 ± 1.1	2.6 ± 1.3	2.4 ± 2.0	0.906	–	–	–
Ocular	4.1 ± 3.1	4.6 ± 3.2	4.4 ± 3.1	3.8 ± 3.0	0.635	–	–	–
Limbs	3.4 ± 1.8	3.4 ± 1.6	3.6 ± 2.1	3.4 ± 1.8	0.925	–	–	–
Gait	8.7 ± 4.2	10.6 ± 4.3	8.2 ± 3.5	8.3 ± 4.3	0.189	–	–	–
Total	26.5 ± 12.6	29.3 ± 11.3	29.3 ± 14.8	24.8 ± 12.3	0.356	–	–	–
BDI	12.6 ± 11.0	9.2 ± 4.8	28.7 ± 10.9	9.0 ± 8.0	< 0.001	> 0.999	< 0.001	< 0.001
Use of antidepressants (%)	16 (22.2)	5 (33.3)	4 (30.8)	7 (14.3)	0.268	–	–	–
MMSE	25.3 ± 2.9	23.8 ± 2.9	25.4 ± 3.2	25.8 ± 2.7	0.077	–	–	–
FAB	8.8 ± 5.2	8.4 ± 4.6	8.5 ± 5.5	9.1 ± 5.5	0.883	–	–	–
PDSS	106.2 ± 34.7	113 ± 21.7	90.1 ± 27.8	108.6 ± 39.0	0.167	–	–	–
NMSS	45.3 ± 45.5	48.4 ± 29.3	96.4 ± 70.6	29.2 ± 26.3	< 0.001	0.296	0.005	< 0.001
PDQ-39 SI	26.7 ± 21.5	31.1 ± 18.7	52 ± 21.8	17.7 ± 15.4	< 0.001	0.035	0.006	< 0.001
SCOPA-AUT	20.1 ± 11.6	21.2 ± 10.7	29.8 ± 12.8	16.8 ± 9.9	0.001	0.518	0.106	0.001

PSP-RS, progressive supranuclear palsy with Richardson’s syndrome; PSP-P, progressive supranuclear palsy with predominant parkinsonism; PSP-PGF, progressive supranuclear palsy with progressive gait freezing; PSP-F, progressive supranuclear palsy with prominent frontal presentation; PFS, Parkinson fatigue scale; PSPRS, progressive supranuclear palsy rating scale; BDI, Beck depression inventory; MMSE, mini-mental state examination; FAB, frontal assessment battery; PDSS, Parkinson’s disease sleep scale; NMSS, non-motor symptoms scale; PDQ-39 SI, Parkinson’s disease questionnaire-39 summary index; SCOPA-AUT, scale for outcomes in Parkinson’s disease-Autonomic.

*p* value < 0.05 considered as the significant.

Bonferroni’s correction was performed to correct for multiple comparisons.

### Differences among the primary, secondary, and non-fatigue patients with PSP

There were no significant differences among the primary, secondary, and non-fatigue groups in terms of age, sex, disease duration, PSP subtype and PSPRS. The BDI of the secondary fatigue group was higher than that of the other groups. The proportion of the patients who taking antidepressants were not differ among the three groups. The MMSE, FAB, and PDSS scores were not different among the three groups. The NMSS of the secondary fatigue group was higher than the primary and non-fatigue group, but there was no difference between the primary and non-fatigue group. In terms of the PDQ-39 summary index (PDQ-39 SI), the secondary fatigue group showed the highest score, followed by the primary and non-fatigue group. Regarding the SCOPA-AUT, differences were only observed between the secondary fatigue and non-fatigue groups (Table 1).

### Logistic regression analysis and correlation between PFS and other clinical scales

We performed logistic regression analysis to determine the factors associated with fatigue in patients with PSP. Results showed that BDI (Odds ratio = 1.143, *p* = 0.001) was significantly associated with fatigue, but other factors did not show a significant association (Table 2).

**Table 2:** Logistic regression analysis of the parameters to identify significantly associated factor with fatigue.

Clinical characteristics	Odds ratio (95% CI)	*p* value
Age	1.120 (1.000–1.254)	0.051
Sex	0.526 (0.122–2.261)	0.388
Disease duration	1.004 (0.984–1.025)	0.676
PSPRS	0.984 (0.923–1.049)	0.622
BDI	1.143 (1.054–1.239)	0.001
MMSE	0.814 (0.643–1.03)	0.086
FAB	1.051 (0.905–1.22)	0.514
PDSS item 15	0.976 (0.769–1.237)	0.838

CI, confidence interval; PSPRS, progressive supranuclear palsy rating scale; BDI, Beck depression inventory; MMSE, mini-mental state examination; FAB, frontal assessment battery; PDSS, Parkinson’s disease sleep scale.

To investigate the relationship between PFS and clinical features, we performed Pearson partial correlation analysis, adjusting for potential confounders, including age, disease duration, and BDI. Total PSPRS score, MMSE, FAB and PDSS were not correlated with PFS scores in patients with PSP. In contrast, NMSS, PDQ-39 SI and SCOPA-AUT were positively correlated with fatigue (Table 3). The sleep/fatigue scale of the NMSS was not correlated with PFS (r = 0.249, *p* = 0.365) (Supplementary Table 2).

**Table 3:** Correlation between Parkinson fatigue scale and other clinical scales.

	r	*p* value
PSPRS	− 0.003	> 0.999
MMSE	− 0.171	> 0.999
FAB	0.150	> 0.999
PDSS	0.212	0.742
NMSS	0.355	0.042
PDQ-39 SI	0.473	0.001
SCOPA-AUT	0.385	0.021

PFS, Parkinson fatigue scale; PSPRS, progressive supranuclear palsy rating scale; MMSE, mini-mental state examination; FAB, frontal assessment battery; PDSS, Parkinson’s disease sleep scale; NMSS, non-motor symptoms scale; PDQ-39 SI, Parkinson’s disease questionnaire-39 summary index; SCOPA-AUT, scale for outcomes in Parkinson’s disease-Autonomic.

Pearson partial correlation analysis adjusting for potential confounders (age, disease duration, Beck depression inventory).

*p* value < 0.05 considered as the significant.

Bonferroni’s correction was performed to correct for multiple comparisons.

## Discussion

Herein, we investigated fatigue and related demographic and clinical features of patients with PSP using the PFS, which is a specified scale for assessing fatigue. We also investigated the relationship between fatigue and other NMS, such as depression, cognition, sleep disturbance, QoL, and dysautonomic symptom in PSP patients with PFS. Our study showed that depression was the factor strongly associated with fatigue in patients with PSP. We divided the patients with fatigue into two groups (primary and secondary) based on the presence of depression, and thereby, we were able to minimize the confounding effects of depressive mood.

Our study revealed that fatigue is the common NMS in patients with PSP (38.8%), but the prevalence of fatigue was much lower than that reported in previous studies (from 82.9 to 100%)^2,6–9^. Chaithra et al*.* investigated the NMS in patients with PSP using NMSS and reported that sleep/fatigue was the most common NMS in these patients, with a prevalence of 82.9%^7^. Ou et al*.* also investigated the NMS in patients with PSP using NMSS and reported that 100% of these patients (27/27) had sleep/fatigue symptoms^6^. However, none of these five studies used the specialized scale for assessing fatigue. Four out of the five studies used sleep/fatigue domains of NMSS^2,6–8^ and the other study used a simple question to investigate fatigue^9^. Although the sleep/fatigue domain of NMSS consists of questions about sleep and fatigue, it might not be sufficient to assess fatigue. Our result, which showed a much lower prevalence of fatigue in patients with PSP, supports this contention. Interestingly, there were no differences in the sleep/fatigue domain of NMSS between the primary and non-fatigue groups, and the sleep/fatigue scale was not correlated with PFS. Only the secondary fatigue group, in which patients had fatigue and depression simultaneously, showed a higher NMSS sleep/fatigue domain score than the primary and non-fatigue groups (Supplementary Table S1 online). Furthermore, fatigue in patients with PSP was not related to sleep quality (PDSS). These results suggest that the sleep/fatigue domain of NMSS might not be useful in precisely detecting fatigue alone in patients with PSP. Contrary to previous studies, we used PFS, which is a specialized scale for the assessment of fatigue. Given the complexity and difficulty in assessing fatigue, our study presents a more reliable result regarding the prevalence of fatigue in patients with PSP.

The pathophysiology of fatigue in neurological diseases, including PD, has not been well studied^3,4^. In PD, fatigue is believed to be an intrinsic symptom rather than a secondary or reactive symptom, because it may precede the motor symptoms and did not correlate with disease duration or motor disability^4,14^. Previous studies investigating fatigue in PD suggest that fatigue is not simply related to the striatal dopaminergic system but is also associated with the imbalance between different neurotransmitters. Studies on PD suggest that the serotonergic system may contribute to the development of fatigue; however, further investigation is needed to explain the pathophysiology^14,15^. As with PD, the present study showed that fatigue in patients with PSP is not associated with disease duration and disease severity (PSPRS). Instead, fatigue in patients with PSP showed a positive correlation with NMSS. Furthermore, a previous study investigating prodromal symptoms of PSP showed that 10% of patients with PSP had fatigue before the diagnosis of PSP, similar to that observed in PD^16^. These results suggest that fatigue in PSP might not only be related to the dopaminergic system but also to the non-dopaminergic system, as in PD. However, there is a paucity of evidence regarding fatigue in PSP; therefore, further studies are required to understand the pathophysiology of fatigue in PSP.

The logistic regression model of the present study showed that BDI play a crucial role in fatigue. Nevertheless, there was no difference in the use of antidepressants among the groups. The results suggest that the effect of taking antidepressants on NMS is minimal, and fatigue and depression may be more important on NMS of PSP. Total of 72 patients, 22 of whom had depression and only 8 of them (36.4%) was taking antidepressant. It suggests that depression in PSP might be undertreated because of the side effects, or the lack of awareness. There are few other possibilities associated with the using the antidepressants. Last, the antidepressants can be used for the behavior control in PSP, not just for the depression as used in frontotemporal lobe dementia in clinical practice^17,18^. Therefore, it is unclear whether all the patients who taking antidepressants had depression or not.

Both the primary fatigue and secondary fatigue groups had worse QoL (PDQ-39 SI) than the non-fatigue group, despite the lack of differences in PSP-related symptoms (PSPRS). Our results suggest that primary and secondary fatigue can worsen the QoL of patients with PSP. Thus, improving fatigue can help to improve the QoL of patients with PSP. Unfortunately, fatigue in parkinsonism is poorly understood, and its treatment is limited^4^. Several medications such as methylphenidate, dopaminergic medications, and antidepressants have been suggested for the treatment of PD-associated fatigue^4^. Generally, patients with PSP exhibit a poor response to dopaminergic replacement therapy, and this therapy has a limited effect on these patients^19^. Given that secondary fatigue patients with depression have worse QoL than non-fatigue patients, antidepressants would be worth trying. The other possibility is that the secondary fatigue patients likely to represent the PSP patients who had higher non-motor burden, not just simply ‘depressive fatigued patients’. However, it needs more investigations to reveal the association between the depression, fatigue, and other NMS in PSP.

Several scales have been introduced for the assessment of fatigue. The PFS is a specialized scale for measuring fatigue in PD patients^20^. However, not all scales, including PFS, have been validated for patients with PSP. We believe that PFS is the most reliable scale for assessing fatigue in patients with PSP, considering the clinical similarity between PSP and PD. Moreover, several scales have been suggested as an assessment tool for PSP in patients with PD, although these scales have not been validated in PSP^21^.

In conclusion, fatigue is a common NMS in patients with PSP and is correlated with other NMS and QoL in these patients. The pathophysiology and clinical impact of fatigue in patients with PSP are largely unknown. The first step in the treatment of fatigue is the identification of symptoms and investigation of the associated factors. In this aspect, the present study provides meaningful insight into fatigue in patients with PSP. Further studies are needed to reveal the pathophysiology of fatigue in patients with PSP and to develop novel treatment modalities.

## Methods

### Participants

Patients with PSP who visited the Movement Disorders Clinic of the Samsung Medical Center between January 2018 and December 2020 were recruited. PSP was diagnosed based on the Movement Disorders Society criteria, and patients with probable PSP were included in the study^1^. The inclusion criteria were: (1) age 40 to 80 years, (2) decreased DAT uptake in the striatal dopaminergic depletion determined using ^18^F-radiolabeled N-(3-fluoropropyl)-2β-carboxymethoxy-3β-(4-iodophenyl) nortropane PET, and (3) Mini-Mental State Examination (MMSE) score ≥ 21^22,23^. Patients who had any of the following conditions were excluded: (1) cancer, hypo- or hyperthyroidism, liver cirrhosis, chronic kidney disease, and (2) history of relevant head injury, cerebrovascular diseases, or musculoskeletal diseases. This study was approved by the Institutional Review Board of the Samsung Medical Center, and all subjects provided written informed consent. All methods were performed in accordance with the relevant guidelines and regulations.

### Clinical assessment

Patient demographics including age and sex, and information on disease duration, diagnosis, and comorbidities were collected. The PSP-rating scale (PSPRS) was used to assess all enrolled patients by movement disorder specialists^24^. Parkinson Fatigue Scale (PFS) was used to assess fatigue^20^. We also assessed the patients using the Beck Depression Inventory (BDI)^11^, MMSE^23^, Frontal Assessment Battery (FAB)^25^, PD sleep scale (PDSS)^26^, non-motor symptom scale (NMSS), PD questionnaire-39 (PDQ-39)^27^, and scale for outcomes in PD-autonomic (SCOPA-AUT)^28^. Fatigue was considered when the mean PFS was ≥ 3.3^20^. The patients were divided into the primary fatigue and secondary fatigue group, referred to the previous study^12^. The fatigued patients who without depression (BDI < 16)^29^ and without sleep disturbance (PDSS > 82)^30^ classified as primary fatigue, and who had any one of two-condition classified as secondary fatigue.

### Statistical analyses

The normality of the data was evaluated using the Shapiro–Wilk test. We included the following covariates in the logistic model: age, sex, disease duration, PSPRS, BDI, MMSE, FAB, and daytime sleepiness (item 15 score of the PDSS)^12,13^. Demographic and clinical features were compared using the analysis of variance (ANOVA) test, chi-square test, or Fisher’s exact test, depending on the variable. We also performed the logistic regression analysis to determine the factors associated with fatigue. The association between PFS and other investigated scales was analyzed using Pearson partial correlation analysis, adjusting for potential confounders (age, disease duration, and BDI). Bonferroni’s correction was performed to correct for multiple comparisons. All tests were two-tailed, and the α level was set to *p* < 0.05. Statistical analyses were performed using IBM SPSS for Windows (version 27.0; IBM Inc., Armonk, NY, USA).

## Supplementary Information

Supplementary Information.
