# Supplementary material for: Understanding fatigue in progressive supranuclear palsy
Source: Sci Rep. 2021 Aug 19;11:16926. doi: 10.1038/s41598-021-96443-z (PMC8376988; doi:10.1038/s41598-021-96443-z)
Supplement: Supplementary file 1 — Supplementary Information. [file 41598_2021_96443_MOESM1_ESM.docx]

**Supplementary Table 1. Differences of the Non-motor symptoms scale, Parkinson’s disease questionnaire 39 summary index, and scale for outcomes in Parkinson’s disease-autonomic subscores among the three groups.**

|  | Primary Fatigue (n = 15) | Secondary Fatigue (n = 13) | Non-fatigue (n = 44) | *p* value- | *p* value (primary vs. non-fatigue) ^1^ | *p* value (primary vs. secondary fatigue) ^1^ | *p* value (secondary vs. non-fatigue) ^1^ |
| --- | --- | --- | --- | --- | --- | --- | --- |
| NMSS |  |  |  |  |  |  |  |
| Cardiovascular including falling | 2.0 (0.0 - 4.0) | 4.0 (1.5 - 10.5) | 0.0 (0.0 - 2.0) | 0.002 | 0.223 | 0.390 | 0.003 |
| Sleep/Fatigue | 6.0 (2.0 - 8.0) | 17.0 (7.0 - 21.5) | 3.0 (0.0 - 6.8) | < 0.001 | 0.384 | 0.003 | < 0.001 |
| Mood/Cognition | 5.0 (3.0 - 12.0) | 10.0 (4.0 - 31.0) | 2.0 (0.0 - 5.0) | < 0.001 | 0.014 | 0.294 | < 0.001 |
| Perceptual problem/hallucination | 1.0 (0.0 - 4.0) | 4.0 (1.0 - 5.0) | 0.0 (0.0 - 0.8) | < 0.001 | 0.057 | 0.324 | < 0.001 |
| Attention/Memory | 4.0 (1.0 - 9.0) | 10.0 (3.5 - 18.0) | 2.0 (0.0 - 3.8) | < 0.001 | 0.038 | 0.216 | 0.001 |
| Gastrointestinal track | 2.0 (0.0 - 11.0) | 4.0 (1.0 - 11.0) | 1.0 (0.0 - 3.0) | 0.103 | - | - | - |
| Urinary | 10.0 (3.0 - 16.0) | 14.0 (7.5 - 26.0) | 3.0 (1.0 - 11.8) | 0.009 | 0.322 | 0.555 | 0.009 |
| Sexual function | 2.0 (0.0 - 15.0) | 3.0 (0.0 - 15.0) | 0.0 (0.0 - 1.0) | 0.018 | 0.054 | > 0.999 | 0.075 |
| Miscellaneous | 2.0 (1.0 - 4.0) | 10.0 (3.0 - 17.0) | 1.0 (0.0 - 6.0) | 0.014 | > 0.999 | 0.018 | 0.010 |
|  |  |  |  |  |  |  | - |
| PDQ-39 |  |  |  |  |  |  |  |
| Mobility | 50.0 (25.0 - 77.5) | 82.5 (51.3 - 100.0) | 17.5 (2.5 - 65) | 0.001 | 0.126 | 0.099 | 0.001 |
| Activities of daily living | 33.3 (25.0 - 66.7) | 62.5 (43.8 - 89.6) | 16.7 (4.2 - 36.5) | < 0.001 | 0.012 | 0.075 | < 0.001 |
| Emotional Well-being | 12.5 (0.0 - 33.3) | 45.8 (18.8 - 81.3) | 4.2 (0.0 - 25.0) | 0.001 | 0.674 | 0.024 | < 0.001 |
| Stigma | 25.0 (0.0 - 37.5) | 43.8 (12.5 - 71.9) | 12.5 (0.0 - 31.3) | 0.031 | 0.760 | 0.294 | 0.027 |
| Social support | 25.0 (0.0 - 50.0) | 41.7 (4.2 - 62.5) | 0.0 (0.0 - 8.3) | 0.001 | 0.035 | > 0.999 | 0.004 |
| Cognition | 18.8 (6.3 - 50.0) | 50.0 (28.1 - 78.1) | 6.3 (0.0 - 23.4) | < 0.001 | 0.034 | 0.156 | < 0.001 |
| Communication | 25.0 (8.3 - 33.3) | 33.3 (12.5 - 75) | 8.3 (0.0 - 39.6) | 0.022 | 0.329 | 0.510 | 0.035 |
| Bodily discomfort | 25.0 (8.3 - 41.7) | 50.0 (33.3 - 66.7) | 4.2 (0.0 - 22.9) | < 0.001 | 0.036 | 0.003 | < 0.001^a^ |
|  |  |  |  |  |  |  |  |
| SCOPA-AUT |  |  |  |  |  |  |  |
| Gastrointestinal | 4.0 (2.0 - 7.0) | 6.0 (3.0 - 13.0) | 3.0 (2.0 - 6.8) | 0.062 | - | - | - |
| Urinary | 6.0 (3.0 - 10.0) | 7.0 (5.5 - 14.0) | 4.0 (2.0 - 6.0) | 0.002 | 0.167 | 0.822 | 0.003 |
| Cardiovascular | 2.0 (1.0 - 3.0) | 3.0 (1.5 - 6.0) | 1.0 (0.0 - 2.0) | < 0.001 | 0.026 | 0.468 | 0.001 |
| Thermoregulatory | 1.0 (0.0 - 3.0) | 2.0 (0.5 - 4.0) | 0.0 (0.0 - 1.8) | 0.037 | 0.385 | 0.948 | 0.039 |
| Pupillomotor | 0.0 (0.0 - 1.0) | 1.0 (0.0 - 1.0) | 0.0 (0.0 - 0.8) | 0.127 | - | - | - |
| Sexual | 6.0 (1.0 - 8.0) | 8.0 (2.5 - 8.0) | 8.0 (1.0 - 8.0) | 0.948 | - | - | - |

NMSS, Non-motor symptoms scale; PDQ-39 SI, Parkinson’s disease questionnaire summary index; SCOPA-AUT, Scale for outcomes in Parkinson’s Disease-Autonomic.

Data expressed as median (interquartile range).

*p* value < 0.05 considered as the significant

Bonferroni’s correction was performed to correct for multiple comparisons.

**Supplementary Table 2. Correlations between the Parkinson fatigue scale and subscore of the various clinical scales.**

|  | r | *p* value- |
| --- | --- | --- |
| NMSS | 0.209 | 0.783 |
| Cardiovascular including falling | 0.238 | 0.458 |
| Sleep/Fatigue | 0.249 | 0.365 |
| Mood/Cognition | 0.067 | > 0.999 |
| Perceptual problem/hallucination | 0.302 | 0.111 |
| Attention/Memory | 0.175 | > 0.999 |
| Gastrointestinal track | 0.216 | 0.686 |
| Urinary | 0.219 | 0.649 |
| Sexual function | 0.070 | > 0.999 |
| Miscellaneous | 0.209 | 0.783 |

NMSS, Non-motor symptoms scale; PDQ-39 SI, Parkinson’s disease questionnaire summary index; SCOPA-AUT, Scale for outcomes in Parkinson’s Disease-Autonomic.

Data expressed as median (interquartile range).

*p* value < 0.05 considered as the significant

Bonferroni’s correction was performed to correct for multiple comparisons.
